# Stress granules affect the dual PI3K/mTOR inhibitor response by regulating the mitochondrial unfolded protein response

**DOI:** 10.1186/s12935-024-03210-x

**Published:** 2024-01-18

**Authors:** Nan Lin, Liankun Sun, Jiannan Chai, Hang Qi, Yuanxin Zhao, Jiaoyan Ma, Meihui Xia, Xiaoqing Hu

**Affiliations:** 1 First Hospital of Jilin University, Changchun, China; 2 Department of Pathophysiology, College of Basic Medical Sciences, Jilin University, Changchun, 130021 China; 3 Department of Clinical Laboratory, First Hospital of Jilin University, Changchun, 130021 China; 4 Department of Obstetrics, First Hospital of Jilin University, Changchun, 130021 China; 5 Department of Ophthalmology, First Hospital of Jilin University, 130021 Changchun, China

## Abstract

Drug resistance remains a challenge in ovarian cancer. In addition to aberrant activation of relevant signaling pathways, the adaptive stress response is emerging as a new spotlight of drug resistance in cancer cells. Stress granules (SGs) are one of the most important features of the adaptive stress response, and there is increasing evidence that SGs promote drug resistance in cancer cells. In the present study, we compared two types of ovarian cancer cells, A2780 and SKOV3, using the dual PI3K/mTOR inhibitor, PKI-402. We found that SGs were formed and SGs could intercept the signaling factor ATF5 and regulate the mitochondrial unfolded protein response (UPR^mt^) in A2780 cells. Therefore, exploring the network formed between SGs and membrane-bound organelles, such as mitochondria, which may provide a new insight into the mechanisms of antitumor drug functions.

**Supplementary Information:**

The online version contains supplementary material available at 10.1186/s12935-024-03210-x.

## Introduction

Ovarian cancer is one of the most lethal cancers in women and, in recent years, the development of drug resistance has led to poor treatment outcomes [1]. Besides cancer cell genetic mutations lead to drug resistance, research findings suggest that the adaptive stress response induced by chemotherapeutic agents may also be one of the major causes of drug resistance [2]. The integrated stress response maintains the adaptive stress response by phosphorylating eIF2α as a key factor that translates genes containing open upstream reading frames (uORFs), such as ATF4, CHOP, and ATF5, re-establishing proteostasis and leading to reduced sensitivity to chemotherapeutic agents [3, 4]. Phosphorylated eIF2α rapidly inhibits mRNA translation by disrupting cap-dependent translation initiation, causing a large number of untranslated mRNAs, RNA-binding proteins, and messenger ribonucleoprotein structures to accumulate in the cytoplasm to form stress granules (SG), which believed to play a role in maintaining the adaptive stress response by rapidly regulating changes in intracellular mRNA translation [5, 6]. Further exploration of the role of SGs in drug resistance in cancer cells has received attention [7, 8].

SGs can regulate untranslated mRNAs, selectively suspending, translating, or degrading mRNAs, and acting as a hub for cell signaling during stress [9]. Somasekharan et al. suggested that YB-1 could promote the synthesis of G3BP1 and therefore induce SGs formation [10], suggesting that RNA-binding proteins (RBPs), such as YB-1, are involved in regulating SGs-internal mRNAs [11, 12]. Recently, many researches confirmed SGs facilitate cancer cells to gain chemoresistance, for example, Timalsina et al. said that SGs are involved in chemoresistance to cisplatin and paclitaxel in human cervical cancer [13], Lin et al. wrote that SGs assembly enhanced the oxaliplatin-resistant in gastric cancer [14], and cisplatin can induce SGs formation in malignant glioma cells [7].

The PI3K/mTOR pathway is often hyper-activated and complexly cross-regulated in ovarian cancer, suggesting that there has a more complex network in molecular signaling crosstalk [15, 16]. For example, inhibition of the mTOR pathway results in suppressing cap-dependent protein translation [17], and 5′ TOP mRNA accumulation in the SGs by the SG-associated protein TIA-1/TIAR, suggesting that the mTOR pathway is involved in SGs formation [18]. On the other hand, SGs inhibit the mTOR pathway by sequestering components of the mTORC1 complex [19]. These results suggest that SGs formation may be related to the mechanism of targeting mTOR antitumor.

Mitochondria, as main organelles for sensing and alleviating stress, are involved in adaptive stress responses in tumor cells [19]. When oncogenic pathways, such as PI3K/mTOR, are disrupted, the mitochondrial unfolded protein response (UPR^mt^) is activated to reduce stress caused by protein accumulation in cells [20]. When mitochondrial function disturbed, ATF5 is selectively translated under stress and enters the nucleus to act as a transcription factor, increasing levels of mitochondria-associated proteases and molecular chaperones, thus establishing new proteostasis and protecting the cell from death signals [21, 22]. Therefore, the translocation of ATF5 is a key factor in activating mtUPR to induce an adaptive stress response [22]. ATF5 enters mitochondria to be degraded under normal conditions and enters the nucleus when cells are stressed, where it is involved in transcriptional activation of the mitochondrial unfolded protein response (UPR^mt^) [23]. Although no experimental studies have reported the correlation of ATF5 with SGs, Souquere et al. found that SGs formed near mitochondria when HeLa and H293 cells were in oxidative stress [24]. Based on the above rationale, we speculate that SGs, as a kind of protection mechanism, can intercept ATF5, causing a change in localization, and then an adaptive stress response is activated, increasing drug resistance. This suggests that it is more important to explore the mechanism of molecular targeted drugs for SGs.

In this study, we used two ovarian cancer cell lines to investigate the mechanism of SGs formation under the dual PI3K/mTOR inhibitor treatment. Also, we elucidated the mechanism of reshaping cellular proteostasis, which SGs regulate mRNA translation and relevant molecules to activate mtUPR.

## Methods and materials

### Cell lines and cell culture

SKOV3 and A2780 ovarian cancer cells were grown in RPMI-1640 (Gibco Life Technologies, USA) supplemented with 10% fetal bovine serum (Invitrogen, USA) at 37 °C in a 5% CO2 concentration.

### Reagents and antibodies

Reagents used in this study include the following: Methylthiazolyldiphenyl-tetrazolium bromide (MTT)(S6821), PKI-402(S2739), cycloheximide (CHX)(NSC-185) and Thapsigargin (Tg) (S7895)were commercially sourced from Selleckchem (Houston, TX, USA), anti-G3BP1 (13057-2-AP), anti-eIF2α (11233-1-AP), anti-4EBP1 (60246-1-Ig), anti-caspase-9(66169-1-Ig), anti-Bcl-2 (26593-1-AP), anti-Bax (50599-2-Ig), anti-Hsp60 (15282-1-AP), anti-Lonp (66043-1-Ig), anti-Clpp (15698-1-AP), anti-Trap1 (10325-1-AP), anti-Actin(HRP-60008), anti-VDAC (10866-1-AP) (Proteintech, Chicago, IL, USA), anti-YB-1(sc-101198), anti-ATF5(sc-377168), anti-p-Akt(Ser473) (sc-101629)(Santa Cruz, CA, USA), p- eIF2α(Ser51)(3398), p-4EBP1(Thr37/46)(2855), p-Akt(Thr308) (13038), p-P70S6K(9204) (Cell Signaling Technology, USA).

### Cell viability assay

Cells were seeded in 96-well plates overnight at a density of 8000 cells/well. The cells were then treated with drugs for 24 h. Cell viabilities were assessed using an MTT assay and absorbance values were measured at 490 nm using a Vmax Microplate Reader (Molecular Devices, USA).

### Flow cytometry

Mitochondrial membrane potential (MMP) and ROS production were determined by JC-1 or DCFH-DA staining (Beyotime Biotechnology, Shanghai, China).

### Western blot assay

Whole-cell lysates were prepared and quantified according to standard protocols. Lysates diluted with 5 × SDS-PAGE loading buffer were boiled at 95 °C for 10 min and separated by SDS-PAGE, and then electrophoretically transferred to polyvinylidene difluoride membranes. The membranes were blocked with 5% milk followed by successive incubation with primary antibodies and peroxidase-conjugated secondary antibodies. The bands were visualized using Pierce ECL Western Blot Substrate (Thermo Scientific, Waltham, MA, USA).

### Immunofluorescence assay

Cells were washed with PBS, fixed with 4% paraformaldehyde for 20 min, and permeabilized with 0.1% Triton X-100 for 8 min. After blocking with 5% bovine serum albumin for 30 min, cells were incubated with primary antibody overnight at 4 °C. After washing with PBS, cells were incubated at room temperature for 1 h in the dark with secondary antibodies conjugated with FITC/Texas Red ((Proteintech Group, Inc, USA). The images were observed on an Echo-lab Revolve microscope (CA, USA).

### RNA Binding Protein Immunoprecipitation (RIP)

RIP was performed as previously described [25] with some modifications. 10^7 cells were harvested by trypsinization and resuspended in 2 ml PBS, wash 2 times and then resuspended in 1 ml RIP buffer [150 mM Kcl, 25 mM Tris pH 7.4, 5 mM EDTA, 0.5 mM DTT, 0.5% NP40, 9 μg/ml leupeptin, 9 μg/ml pepstatin, 10 μg/ml chymostatin, 3ug/ml aprotinin, 1 mM PMSF. Resuspended cells were split into a fraction of 300 μl (for IP), then centrifugated at 13,000 RPM for 10 min. Add antibody YB-1 to supernatant and incubated for 2 h at 4 °C. 40 μl of protein A/G beads were added and incubated for 4 h at 4 °C with gentle rotation. Beads were pelleted at 2500 RPM for 30 s, the supernatant was removed and beads were resuspended in 500 μl RIP buffer and washed 3 times in RIP washes, then wash 1 time in PBS. Beads were resuspended in 1 ml of Trizol. Co-precipitated RNAs were isolated and RT-PCR for G3BP1(forward 5′-ATGCAGTCTACG- GACAGAAAGA-3′ and reverse 5′-GAGCATCAACATGGCGAATCT-3′).

### Immunoprecipitation assay

Cells were lysed in NP40 lysis buffer. Equal amounts of lysates were immunoprecipitated with 2 μg of p62 antibody overnight at 4 °C. Then 25 μL of protein A and G agarose (Beyotime, China) was then used for each sample. The beads were washed with PBS three times with 1 ml each. The eluted proteins were examined by western blotting.

### Nuclear and mitochondrial isolation

Cells were harvested after treatment with various reagents. Nuclear and mitochondrial fractions were extracted using a nuclear fractionation kit and a mitochondrial fractionation kit, respectively (Beyotime Technology).

### Statistical analysis

All experimental data represent at least 3 independent experiments and were presented as mean ± standard deviation (SD) and were carried out using the Student's t-test. P < 0.05 were considered statistically significant difference. Statistical analysis was performed with GraphPad Prism 7.0 (La Jolla, CA).

## Result

### Adaptive stress response was activated in A2780 cells

**Fig. 1:**
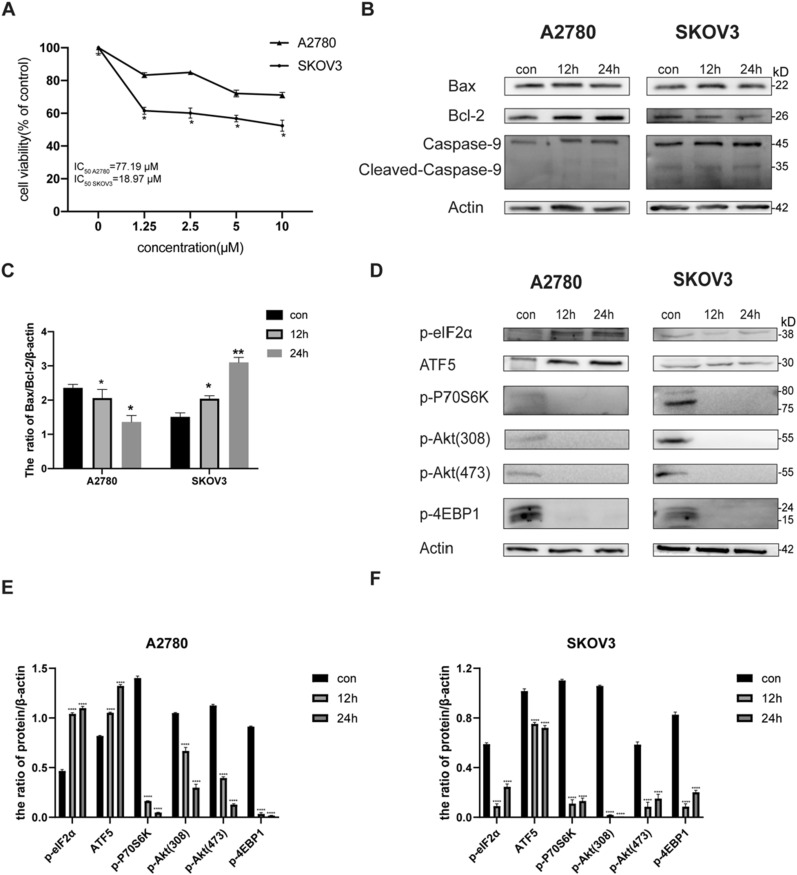
Adaptive stress response was activated in A2780 cells. **A** A2780 and SKOV3 cells were treated with 1.25–10 μM PKI-402 for 24 h and then cell viability was detected by MTT assays, and IC50 was calculated by GraphPad Prism 7.0. **B** A2780 and SKOV3 cells were treated with PKI-402 (2.5 μM) for 12 h and 24 h, respectively, and levels of apoptosis-associated proteins were detected by western blotting. **D** A2780 cells and SKOV3 cells were treated with PKI-402 (2.5 μM) for 12 h. Cell lysates were immunoblotted using the indicated antibodies. **C**, **E**, **F**. Levels of the proteins in **B** and **D** were measured by western blotting, respectively. Data were expressed as mean ± SEM of three independent experiments; ****P < 0.0001 compared to the control

In ovarian cancer cells, the *PIK3CA* gene that encodes type I PI3Ks P110α is frequently mutated, which is the reason for the reduced sensitivity of tumor cells to chemotherapeutic drugs [26]. We found that two types of ovarian cancer cells, SKOV3 and A2780, showed different sensitivities in the treatment of the dual targeting inhibitor PKI-402. The mutation site in SKOV3 cells has been observed to be H1047R in the PI3Kα kinase structural domain of the PI3K kinase, while the mutation site in A2780 cells is E365K in the structural domain C2, which may be the reason for the difference in sensitivity of the two cells to the dual targeting inhibitor PKI-402 [27]. Under PKI-402 treatment, the viability of SKOV3 cells decreased (Fig. 1A), the ratio of Bax/BCL-2 increased, and cleaved caspase9 increased too (Fig. 1B, C). Overall, PKI-402 induced to the apoptosis of SKOV3 cells, and compared with A2780, SKOV3 cells were more sensitive to PKI-402.

Interestingly, when we inhibited thePI3K/mTOR pathway, both of phosphorylated eIF2α and ATF5 were obviously increased in A2780 cells (Fig. 1D).

### Stress granules formed in A2780 cells under the treatment of PKI-402

**Fig. 2:**
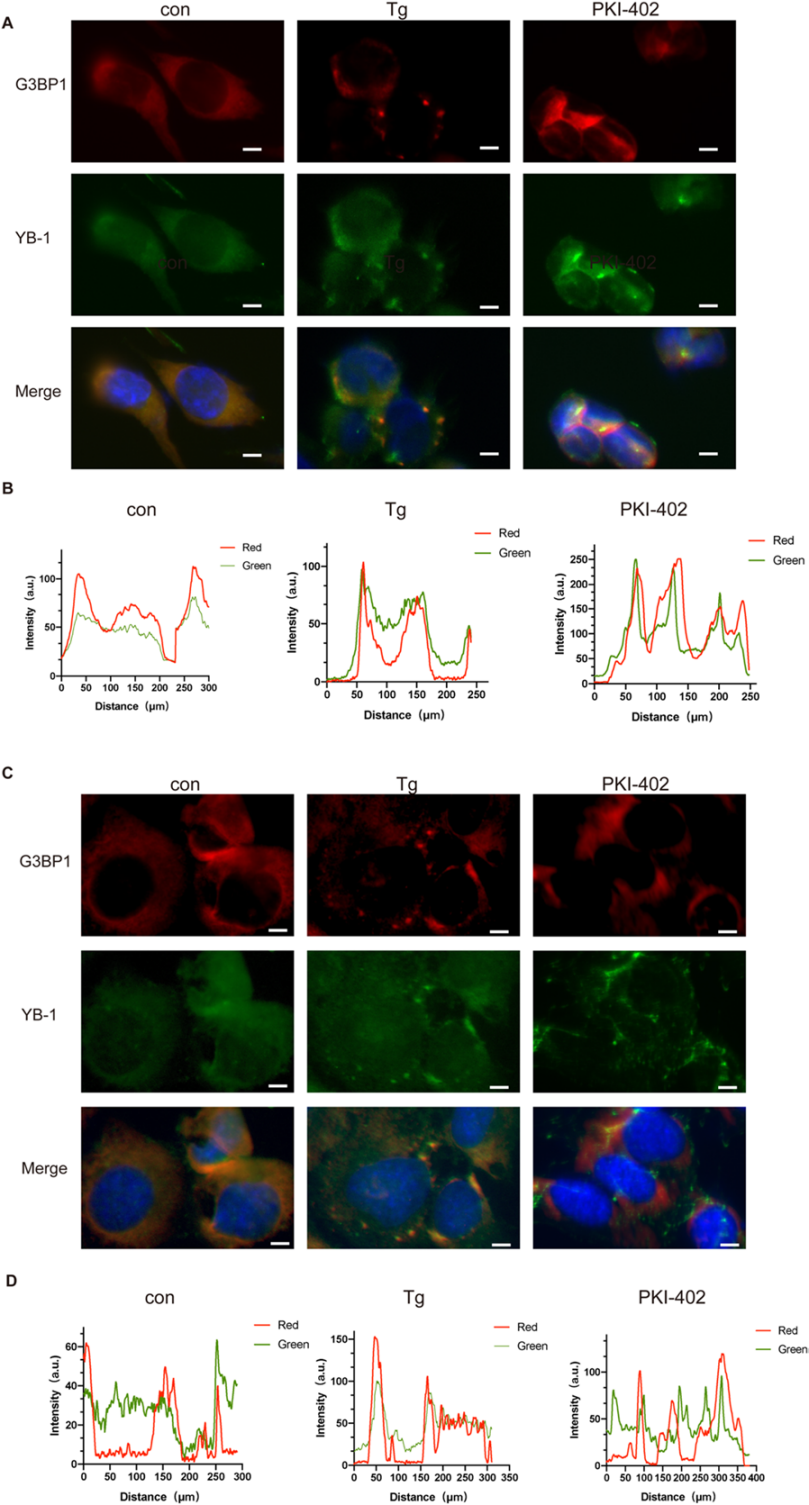
Stress granules form in A2780 cells under the treatment of PKI-402. A.A2780 cells were treated with PKI-402(2.5 μM) for 12 h and thapsigargin (Tg) 1 μM for 50 min. The colocalization of YB-1 and G3BP1 was determined by staining and observed by fluorescence microscopy, scale bar, 10 µm. **B** Colocalizations of YB-1 and G3BP1 in **A** were quantified using ImageJ software. **C** SKOV3 cells were treated as same as A2780 cells. **D** Colocalizations of YB-1 and G3BP1 in **C** were quantified using ImageJ software

PKI-402 increased the level of phosphorylated eIF2α in A2780 (Fig. 1D). Phosphorylated eIF2α leads to a global translational arrest, at which point large amounts of untranslated mRNA, RNA-binding proteins, and messenger ribonucleoprotein complexes undergo liquid phase separation in the cytoplasm to form stress granules [28]. Immunofluorescence assay staining for G3BP1 and YB-1, which are marker proteins of SGs [9], revealed that SGs appeared in A2780 cells which treated by PKI-402 for 12 h (Fig. 2A), while no stress granules formed in SKOV3 cells (Fig. 2C).

### UPR^mt^ was activated and mitochondrial function was improved in A2780 cells

**Fig. 3:**
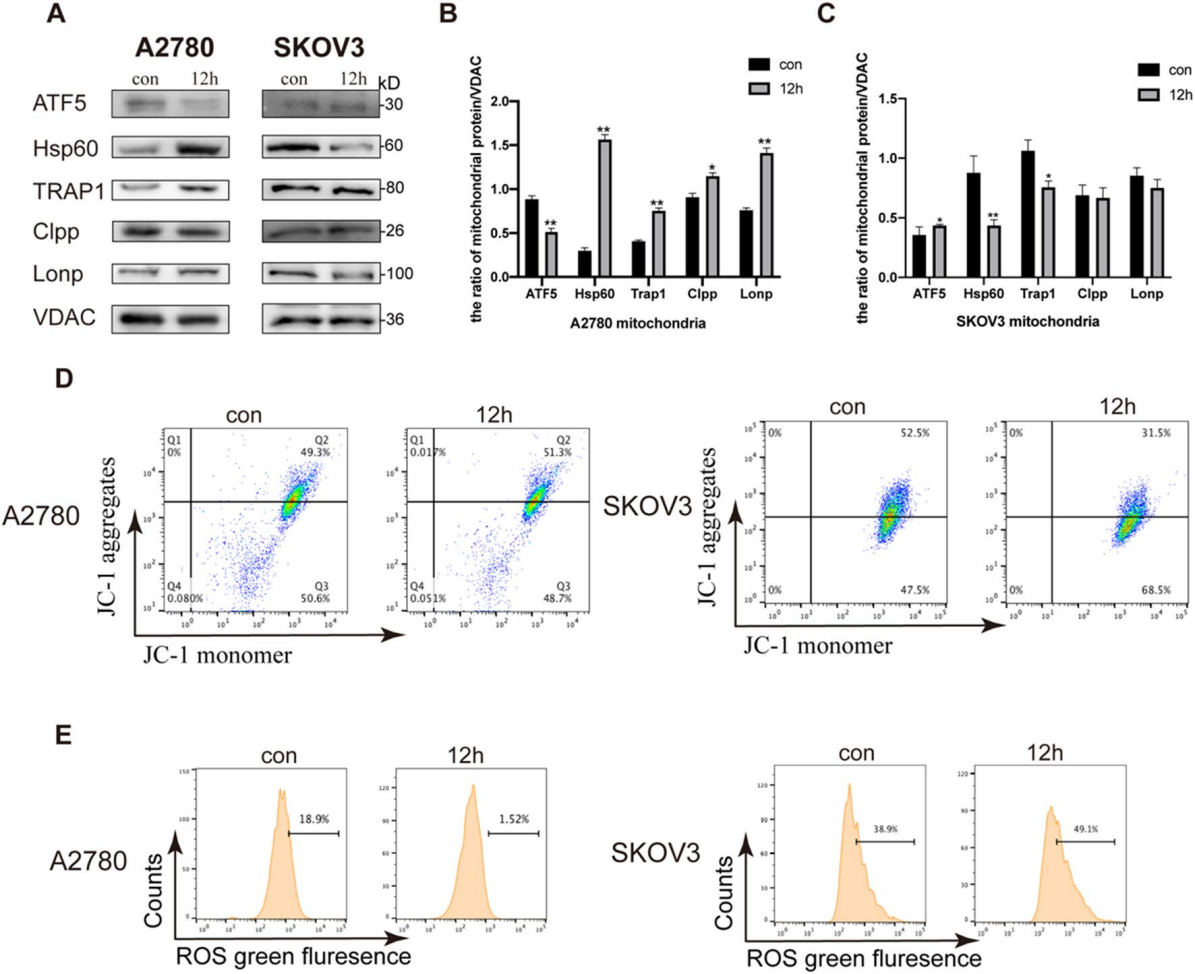
UPR^mt^ was activated and mitochondrial function was enhanced in A2780 cells. **A** A2780 and SKOV3 cells were treated with PKI-402 (2.5 μM) for 12 h, mitochondrial proteins were collected and mitochondrial protease expression and molecular chaperone expression were analyzed by Western blotting. **B** The expression of the proteins in **A** were measured by Western blotting, respectively. Data were expressed as mean ± SEM of three independent experiments; *P < 0.05, **P < 0.01 compared to the control. **D** A2780 and SKOV3 cells were treated with PKI-402 (2.5 μM) for 12 h and then incubated with JC-1. The changes in fluorescence intensity were measured by flow cytometry. **E** A2780 and SKOV3 cells were treated with PKI-402 (2.5 μM) for 12 h and then incubated with DCFH-DA. The changes in fluorescence intensity were measured by flow cytometry

Under PKI-402 treatment, we found ATF5 increased in A2780 cells (Fig. 1D). Next, we isolated mitochondria from two ovarian cancer cells and found that mitochondrial proteases (Clpp and Lonp) and molecular chaperones (Hsp60 and Trap1) increased in A2780 cells compared to SKOV3 cells (Fig. 3A). Lonp and Clpp are ATP-dependent proteases, which can maintain mitochondrial proteostasis [29]. When the accumulation of protein aggregates in mitochondria and produces a mitochondrial stress protein response, mitochondrial molecular chaperones increase to protect mitochondria from stress [30]. Above all, our results confirmed that UPR^mt^ was activated in A2780 cells. Then we examined the mitochondrial membrane potential of both cells and found that the mitochondrial membrane potential was elevated in A2780 (Fig. 3D); we examined intracellular ROS levels and found that ROS was reduced in A2780 cells (Fig. 3E). We also tested relative ATP level and oxygen consumption (Additional file 1: Fig. S1C–F). Based on these results, we concluded that in A2780 cells UPR^mt^ was activated and mitochondrial function was enhanced. On the contrary, there was no significant change in SKOV3 cells.

### The sensitivity of A2780 cells to PKI-402 was increased and UPR^mt^ was inhibited after inhibiting SGs

**Fig. 4:**
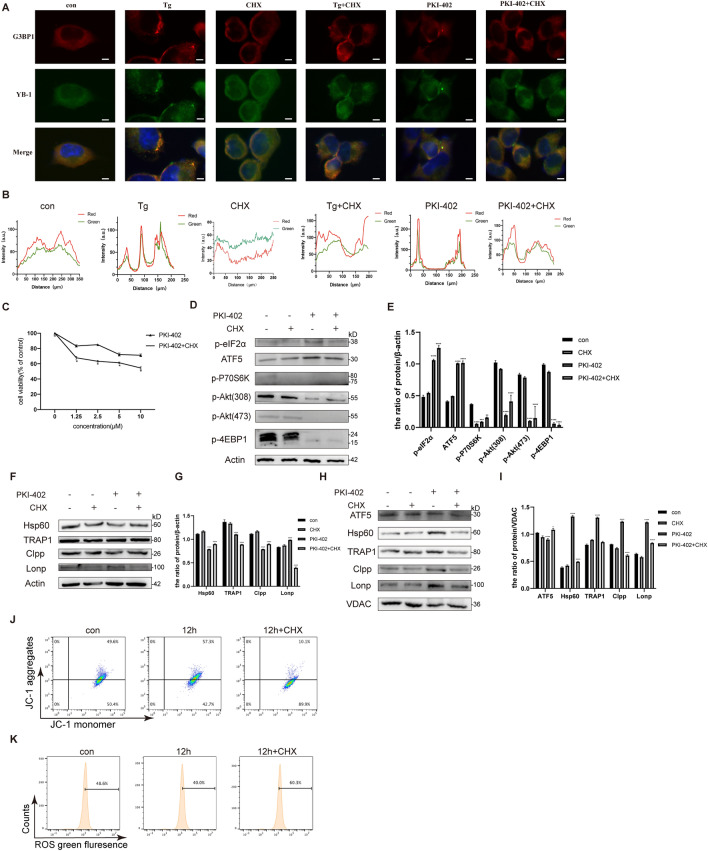
The sensitivity of PKI-402 was increased and mtUPR was inhibited after inhibiting SGs**. A** After treatment with cycloheximide (CHX) 5 μg/ml for 45 min, washed twice with PBS, treated with PKI-402 (2.5 μM) for 12 h, and thapsigargin (Tg) 1 μM for 50 min. Colocalizations of YB-1 and G3BP1 was determined by staining and observed by fluorescence microscopy, scale bar, 10 µm. **B** Colocalizations of YB-1 and G3BP1 in C were quantified using ImageJ software. **C** After CHX treatment, PKI-402 (2.5 μM) was added for 12 h, and then cell viability was detected by MTT assays. **D**, **F** After CHX treatment, PKI-402 (2.5 μM) was added for 12 h, cell lysates were immunoblotted using the indicated antibodies. **H** Same treatment with **D**, **F**, then mitochondrial proteins were collected and the expression of mitochondrial-associated protease and chaperone was analyzed by Western blotting. **E**, **G**, **I** Levels of the proteins in **D**, **F**, and **H** were measured by Western blotting, respectively. Data were expressed as mean ± SEM of three independent experiments; **P < 0.01, ****P < 0.0001 compared to control. **J**, **K** After CHX treatment, PKI-402 (2.5 μM) was added for 12 h, and A2780 cells were treated with PKI-402 (2.5 μM) for 12 h and then incubated with DCFH-DA and JC-1. Changes in fluorescence intensity were measured by flow cytometry

To investigate whether SGs regulate UPR^mt^, we used CHX to inhibit the formation of SGs. Cycloheximide (CHX), a classic protein translation inhibitor and also a specific inhibitor of stress granules [28]. In immunofluorescence experiments, we observed that SG formation was inhibited after CHX treatment for 45 min followed by PKI-402 (Fig. 4A) and that the sensitivity of A2780 cells to PKI-402 was increased (Fig. 4C). Then we found that inhibition of SGs resulted in phosphorylated eIF2α and ATF5 decreased (Fig. 4D), but the overall expression of proteins associated with the mitochondrial unfolded protein response did not change obviously (Fig. 4F). Then, we isolated mitochondria and found a reduced expression of associated proteases and molecular chaperones (Fig. 4H), suggesting that only mitochondrial protein decreased after inhibiting SGs, indicating that there are some connections between SGs and UPR^mt^. Detecting mitochondrial membrane potential and ROS levels, we found A2780 cells showed a decrease in mitochondrial membrane potential (Fig. 4J) and an increase in ROS (Fig. 4K). We also tested ATP level and oxygen consumption rates (Additional file 1: Fig. S1C, E). It was found that when SGs were formed simultaneously, UPR^mt^ activated and mitochondrial function enhanced under the treatment of PKI-402; and we speculated that SGs might regulate mtUPR.

### SGs regulated UPR^mt^ by affecting the localization of ATF5

**Fig. 5:**
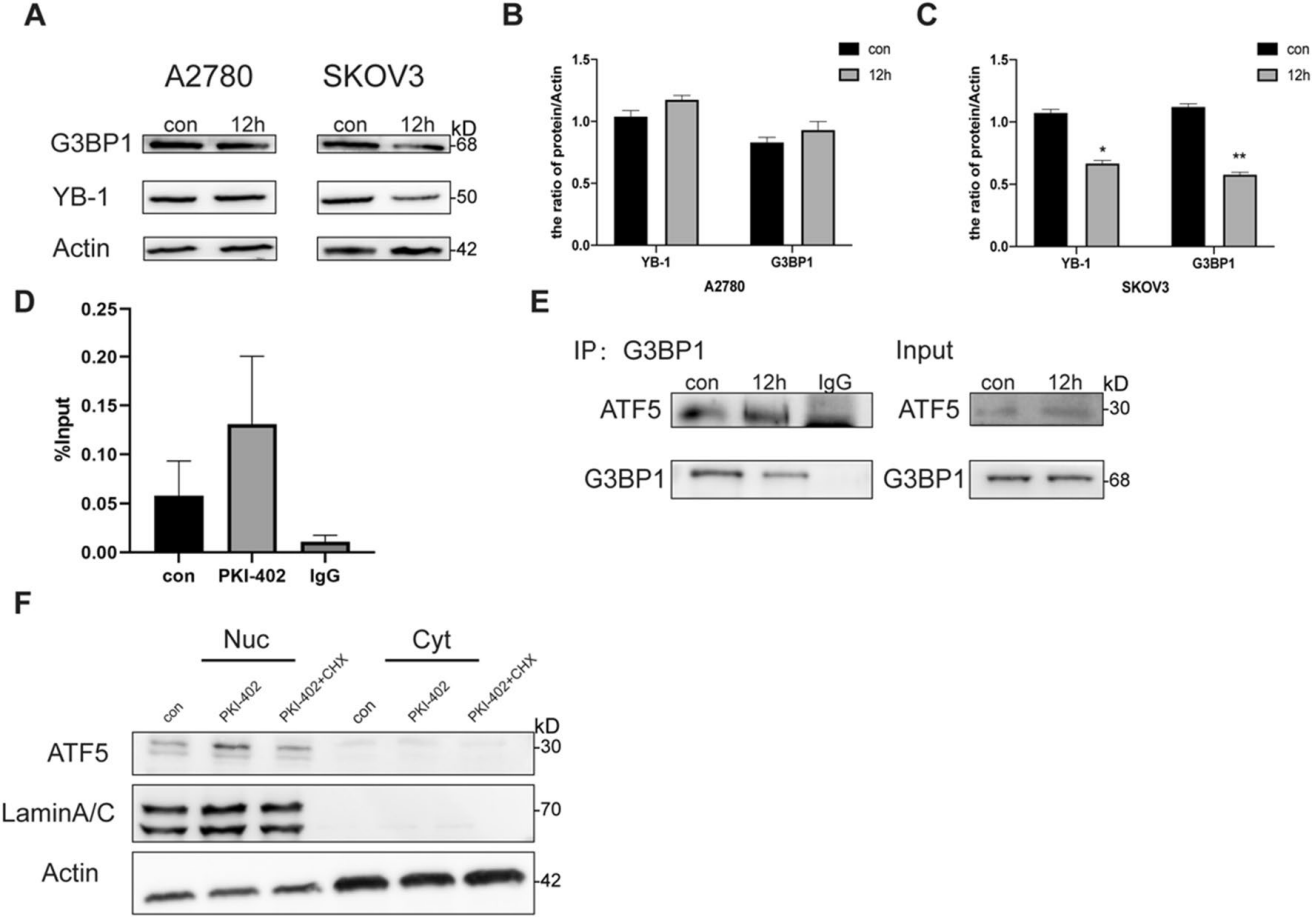
SGs regulate UPR^mt^ by affecting the localization of ATF5. **A** A2780 cells were treated with PKI-402 (2.5 μM) for 12 h. Cell lysates were immunoblotted using the indicated antibodies. **B**, **C**. Levels of proteins in 6A were measured by Western blotting. **D** A2780 cells were treated with PKI-402 (2.5 μM) for 12 h. Cell lysates were obtained using anti-YB-1 and subjected to semiquantitative qPCR using G3BP1. E. Immunoprecipitation was performed using anti-G3BP1 antibody followed by Western blotting using anti-G3BP1 and anti-ATF5 antibodies. **F** Nuclear proteins from A2780 cells were collected after treatment with PKI-402 (2.5 μM) for 12 h and ATF5 was measured by Western blotting

**Fig. 6:**
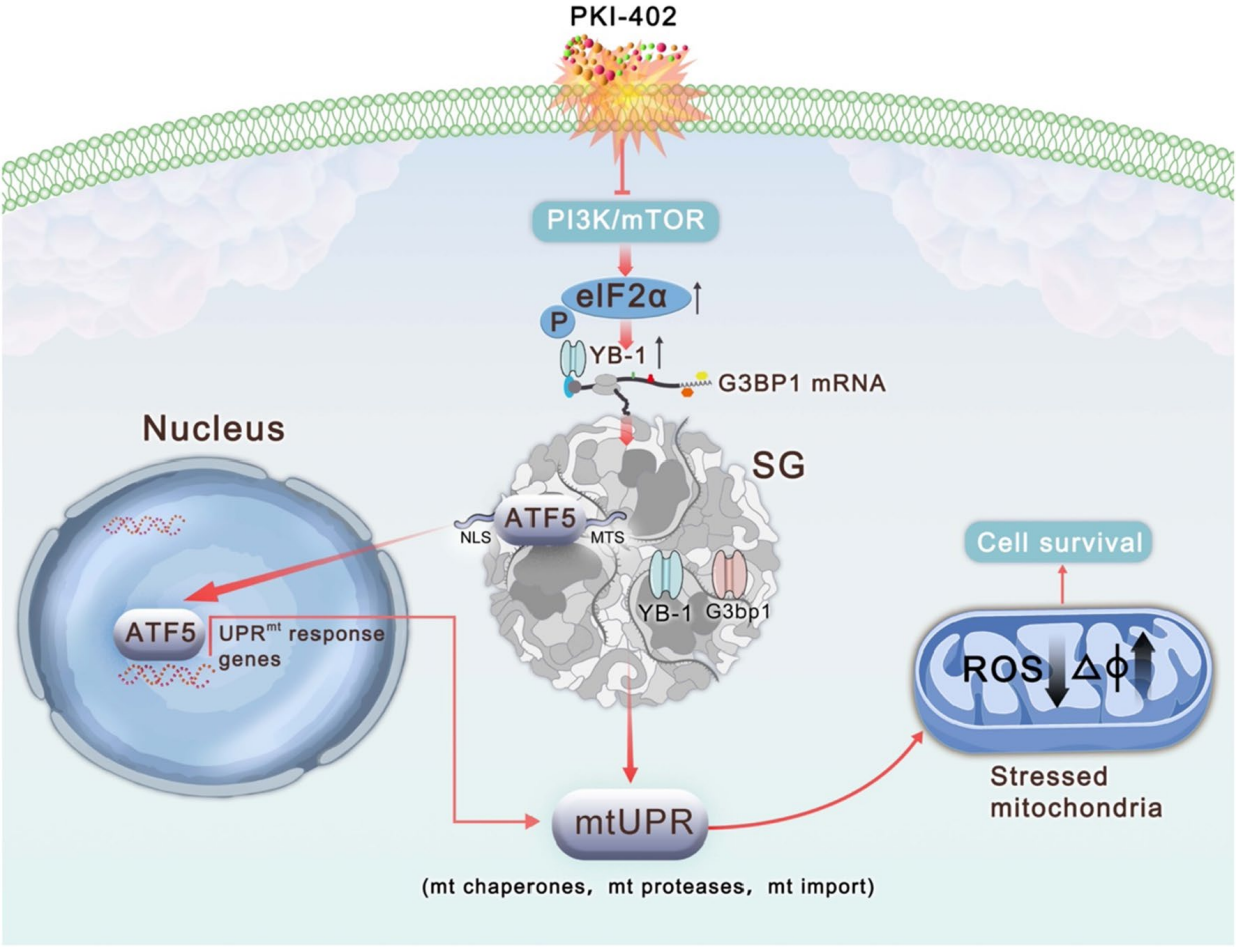
Schematic of proposed mechanisms for the treatment of the dual PI3K/mTOR inhibitor PKI-402, which induces the formation of SGs in A2780 cells and intercepts ATF5, which entered nucleus to regulate mitochondrial unfolded protein response

Since YB-1 has been reported to regulate G3BP1 and induce SGs formation [10], our results showed that after 12 h of treatment with PKI-402, YB-1 and G3BP1 was reduced in SKOV3 cells compared to A2780 cells (Fig. 5A). We performed RNA immunoprecipitation assays and found (Fig. 5D) that YB-1 interacted with mRNAs of G3BP1 in A2780 cells in the presence of PKI-402. In immunoprecipitation experiments (Fig. 5E), showed that G3BP1, the signature protein of SGs, interacted with ATF5. According to functions of SGs, which can recruit signaling molecules [31], we speculated that as two key proteins of SGs, YB-1 and G3BP1 weather paly an important role in ATF5, which could activate UPR^mt^, and SGs protected ATF5 and kept it out of mitochondria after adding dual target inhibitors. Furthermore, we isolated the cytosolic protein and the results showed a clear entry of ATF5 into the nucleus, and when we inhibited SGs, the entry of ATF5 was decrease (Fig. 5F). In order to evaluate the activity of ATF5, we detected the expression of mitochondrial proteases (Clpp and Lonp) and molecular chaperones (Hsp60 and Trap1), results showed expressions of these genes were increased (Additional file 1: Fig. S1G) when treated by PKI-402, while expressions were decreased by CHX and PKI-402. Combining these results, we concluded that the insensitivity of A2780 to PKI-402 is due to SG formation, which alters the location of ATF5, a key molecule in UPR^mt^ [22]. ATF5 entered the nucleus and initiate UPR^mt^, followed by increased levels of proteases and molecular chaperones in mitochondria, and improved mitochondrial function, ultimately leading to the resistance of the dual PI3K/mTOR inhibitor (Fig. 6).

## Discussion

Currently, two main causes of high mortality and morbidity in ovarian cancer are late diagnosis and drug resistance [32, 33]. Aberrant activation of oncogenic pathways, such as PI3K/mTOR, in ovarian cancer cells is key to tumor drug resistance [15]. However, Shen et al. found that translational remodelling of mRNA could cause non-genetic resistance in melanoma, suggesting that selective translation of mRNA by adaptive stress responses is emerging as a major reason of drug resistance [34].

The PI3K/mTOR pathway is important for metabolism, growth, and mRNA translation [17], and is often over-activated in many cancer cells, we can inhibit this signaling pathway to prevent cancer cell growth. Single PI3K/mTOR target inhibitors can easily activate other loops in this pathway [35]. Dual PI3K/mTOR inhibitors are effective in inhibiting the signaling pathway, but after using the dual PI3K/mTOR inhibitor, we found that A2780 cells developed a more complex adaptive stress response, with phosphorylation of eIF2α increasing and accumulation of polyribosome catabolism mRNA appearing as a liquid phase separation, leading to SGs formation [36, 37]. The relationship between mTOR and stress granules is very complicated. On the one hand, it has been reported that inhibiting mTOR can promote the formation of stress granules [38]. Fournier MJ, et al. reported that mTOR/4EBP1/eIF4E axis enhances the ability of SGs assembly [8]. The main reason for this difference may be because of the different environment or the different stress responses activated within the cells. Now, there is a dynamic balance between stress granules and mTOR in the tumor cell. Stress granules can prevent the over-activation of mTOR. On the contrary, mTOR can regulate the formation of stress granules through its downstream molecules, which leads tumor cells to resist to external stress. Therefore, exploring the relationship between stress granules and mTOR is still a focus [39]. Langdon et al. showed that mTOR signaling was inhibited at an early stage with a dual PI3K/mTOR inhibitor, indicating that the dual-targeted inhibitor only prevented cancer progression, and cancer cells still remained viable and could not be treated [40]. Above all, it is reasonable to speculate that SGs formation may be the main reason for tumor cell insensitivity to the dual PI3K/mTOR inhibitor.

Many researches have examined the connection between cancer and SGs, SG-related molecules were up-regulated in hepatocellular carcinoma [41], pancreatic cancer, [42], and malignant glioma [7]. Experiments indicated that using sorafenib in human leukemia cells induces the formation of SGs and increases the resistance of chemotherapeutics [41]. Our results showed A2780 cells were insensitive to the dual PI3K/mTOR inhibitor (Fig. 1A) and intracellularly formed SGs (Fig. 2A). After the addition of the classic SGs inhibitor cycloheximide (CHX) (Fig. 4A) [43], the sensitivity of the dual PI3K/mTOR inhibitor increased obviously (Fig. 4B), all of which verified that SGs influence drug resistance.

In Fig. 5, we discussed the mechanism of SGs formation. After treatment with PKI-402, levels of YB-1and G3BP1 were increased in A2780 cells (Fig. 5A), and we also verified that YB-1 promoted G3BP1 synthesis (Fig. 5D). YB-1 can regulate the stability and translation of mRNAs and also be involved in drug resistance [44]. Therefore, RBP acts as a scaffold, as well as an important functional protein in SGs [6]. When the mTOR pathway is inhibited, a large number of mRNAs accumulate, and RBPs bind them to form a dynamic pool of mRNAs [45]. Depending on cellular needs, RBPs shuttle between SGs and polyribosomes to selectively translate mRNAs, which reshape cellular proteostasis [46, 47]. The interaction between RBPs not only verifies SGs formation but also interact with other molecules to regulate signaling pathway, like adaptive stress response in tumor cells.

When the mTOR pathway inhibited, mitochondrial-associated protein translation was restricted, leading to a large amount of unfolded or misfolded proteins accumulated in mitochondria, which causes stress and initiates mtUPR to prevent mitochondrial dysfunction [23, 48]. In *C. elegans*, the main factor activates UPR^mt^ is ATFS-1, which harbors both a mitochondrial targeting sequence and a nuclear localization sequence, whereas activation of UPR^mt^ is much more complex in mammals [21]. During the stress, molecules containing uORFs, such as ATF4, CHOP, and ATF5, can be selectively translated independently of phosphorylated eIF2α [49]. All three molecules are key to the integrated stress response and are involved in mtUPR. However, Fiorese et al. showed that ATF5 is a key factor in the initiation of UPR^mt^ [21]. Therefore, ATF5 translocation is critical for initiating UPR^mt^. Studies indicated that when mitochondrial stress occurs, the efficiency of mitochondrial input is reduced, resulting in the inability of ATF5 to enter the mitochondria [50, 51]. However, there are other effects on the translocation process. According to our results, ATF5 obviously entered the nucleus (Fig. 5F, Additional file 1: Fig. S1G) and had an interaction with G3BP1 (Fig. 5E), a core component of SG [52]. SGs can isolate specific signaling factors (such as RACK1 [53], HuR, and Lin28 [54]) and integrate multiple stress signaling cascades to coordinate cellular response to counter stress. So, we speculate that ATF5, an important signaling molecule for communication between SGs and mitochondria, is segregated into SGs before entering the nucleus. D'Amico et al. suggested that SGs act as translation hubs to regulate mitochondrial-related translation [20]. When the stress occurs, cells establish a signaling network with SGs as the centre and mitochondria as effectors to resist stress and reshape cellular proteostasis.

In conclusion, we exploreed that during the stress, SGs replaced the translational function of mTOR and became the center of translational regulation, communicating with mitochondria and other membrane-bound organelles, allowing cells to effectively resist and adapt to the stress. Furthermore, targeting this network for interference provides us new ideas for mechanisms of antitumor drugs.

### Supplementary Information

**Additional file 1: Figure S1.** The sensitivity of PKI-402 has correlation with SGs in PC3 cells and the formation of SGs influenced mitochondrial functions in A2780 cells. **A** PC3 cells were treated with 0.125-10 μM PKI-402 for 24 h and then cell viability was detected by MTT assays, and IC50 was calculated by GraphPad Prism 7.0. B. PC3 cells were treated with PKI-402(2.5 μM) for 12  h and thapsigargin (Tg) 1 μM for 50 min. The colocalization of YB-1 and G3BP1 was determined by staining and observed by fluorescence microscopy, scale bar, 10 µm. **C**, **D**. A2780 and SKOV3 cells were treated by PKI-402 for 12 h or CHX for 45 min were detected relative ATP levels, and **E**, **F**. The oxygen consumption rates of 12 h were measured in A2780 cells in the presence of PKI402 and CHX. **F** detected relative the oxygen consumption. **G** A2780 cells were treated with PKI402 and CHX, the expressions of Clpp, Lonp, Hsp60 and Trap1were detected by qPCR.

## Data Availability

All data included in this study are available from the corresponding author upon reasonable request.
