# Supplementary material for: Stress granules affect the dual PI3K/mTOR inhibitor response by regulating the mitochondrial unfolded protein response
Source: Cancer Cell Int. 2024 Jan 18;24:38. doi: 10.1186/s12935-024-03210-x (PMC10795350; doi:10.1186/s12935-024-03210-x)
Supplement: Supplementary file 1 — Additional file 1: Figure S1. The sensitivity of PKI-402 has correlation with SGs in PC3 cells and the formation of SGs influenced mitochondrial functions in A2780 cells. A PC3 cells were treated with 0.125-10 μM PKI-402 for 24 h and then cell viability was detected by MTT assays, and IC50 was calculated by GraphPad Prism 7.0. B. PC3 cells were treated with PKI-402(2.5 μM) for 12 h and thapsigargin (Tg) 1 μM for 50 min. The colocalization of YB-1 and G3BP1 was determined by staining and observed by fluorescence microscopy, scale bar, 10 µm. C, D. A2780 and SKOV3 cells were treated by PKI-402 for 12 h or CHX for 45 min were detected relative ATP levels, and E, F. The oxygen consumption rates of 12 h were measured in A2780 cells in the presence of PKI402 and CHX. F detected relative the oxygen consumption. G A2780 cells were treated with PKI402 and CHX, the expressions of Clpp, Lonp, Hsp60 and Trap1were detected by qPCR. [file 12935_2024_3210_MOESM1_ESM.docx]

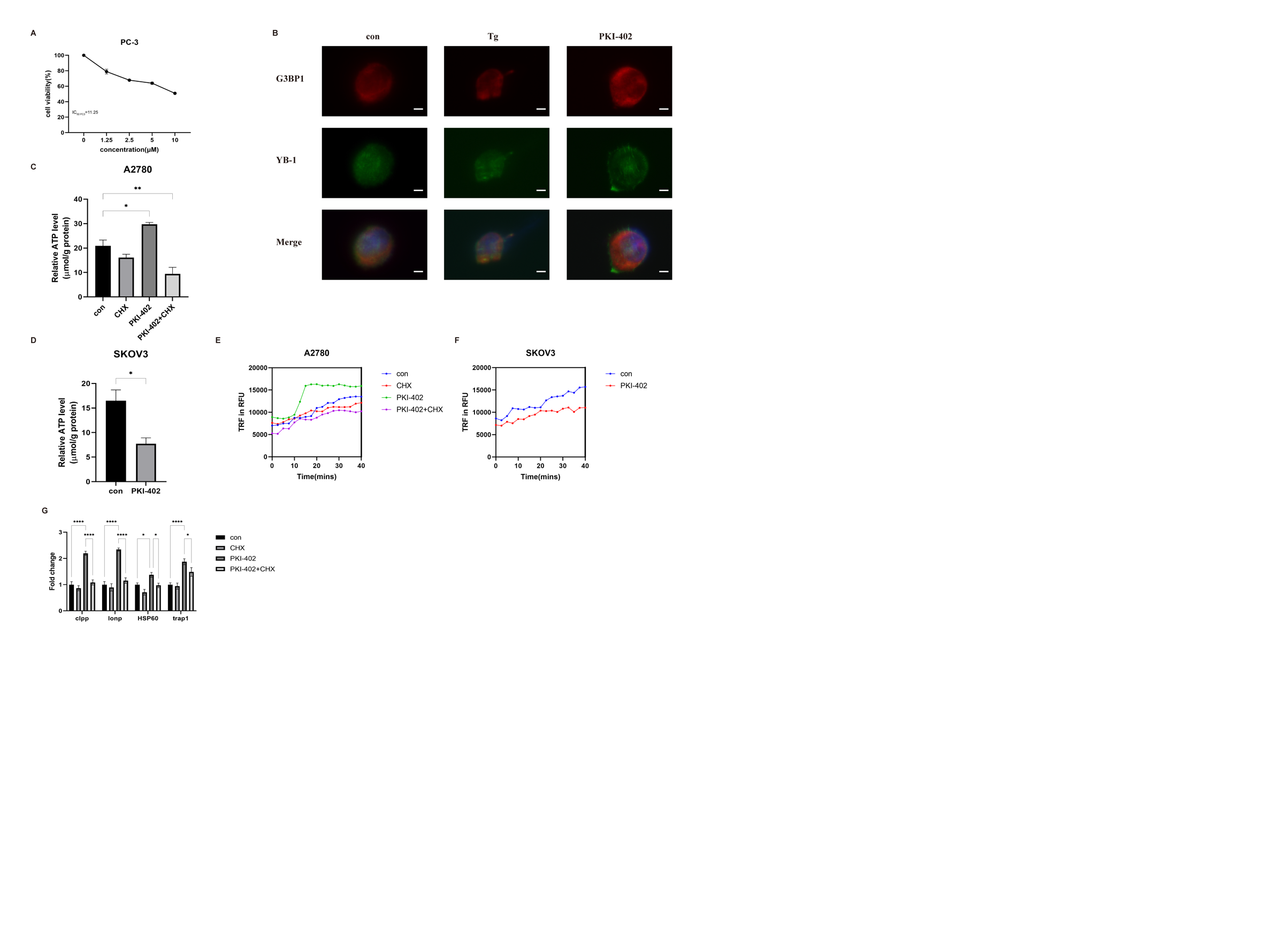


S1. A. PC3 cells were treated with 0.125-10 μM PKI-402 for 24 h and then cell viability was detected by MTT assays, and IC50 was calculated by GraphPad Prism 7.0. B. PC3 cells were treated with PKI-402(2.5 μM) for 12 h and thapsigargin (Tg) 1μM for 50 min. The colocalization of YB-1 and G3BP1 was determined by staining and observed by fluorescence microscopy, scale bar, 10 µm. C, D. A2780 and SKOV3 cells were treated by PKI-402 for 12h or CHX for 45 min were detected relative ATP levels, and E, F. The oxygen consumption rates of 12h were measured in A2780 cells in the presence of PKI402 and CHX. F. detected relative the oxygen consumption. G. A2780 cells were treated with PKI402 and CHX, the expressions of Clpp, Lonp, Hsp60 and Trap1were detected by qPCR.
